# Induction of oral tolerance for cow’s milk proteins. Is everything under control?

**DOI:** 10.1186/2045-7022-3-S3-P17

**Published:** 2013-07-25

**Authors:** C Muñoz Archidona, SJ Quevedo Teruel, JF Viada Bris, T Bracamonte Bermejo, LA Echeverría Zudaire

**Affiliations:** 1 Pediatrics, Hospital Severo Ochoa, Leganés, Madrid, Spain

## Background

Induction of oral tolerance (IOT) represents a new treatment for cow´s milk proteins (CMP) allergy. Several studies show that it is safe and effective in most of cases,but do we have controlled all the risks and complications?

## Methods

We evaluated 59 patients with IgE-mediated allergy for CMP on whom we administered IOT treatment according to our protocol. Initial induction phase was made at hospital (up to 2.5ml), followed by a maintenance phase at home, with an increase of the weekly dosage that was performed in the outpatient clinic. Once a dose of 200ml/day was achieved, continued daily dose was taken at home. We did a clinical follow-up at 6 months and then annually, with skin prick tests and specific IgE levels. We pay special attention to the problems and complications that arose during the IOT and its subsequent follow-up.

## Results

We completed IOT in 58 of 59 patients. Initial IgE specific value for casein was 10.7 KU/L (median). Duration of IOT: 117±47 days (mean). 39% had reactions during induction phase, 47.5% in weekly hospital visit and 83.1% at home (but only 10% were severe). 43 patients were followed for at least one year, 20.3% of them had adverse reactions (most mild reactions). One patient had anaphylaxis after ingesting ibuprofen with cow's milk. Another patient is taking omalizumab for severe asthma. 4 (9% of total) presented digestive symptoms and were subsequently diagnosed of eosinophilic esophagitis. All patientsfeel they have improved their quality of life and no one of them is willing to stop eating CMP despite adverse effects described and even 2 patients began after one year, IOT for egg protein. There was asignificant decrease in specific IgE levels and prick test values one year later of CMP IOT.

## Conclusion

IOT is a safe treatment for CMP allergy with very good results. However, we do not know the outcomes of these patients in the long term and we need close monitoring to prevent adverse events or treat if necessary. Even so, patients generally want to keep taking cow´s milk due to the benefits obtained in their quality of life.

## Disclosure of interest

None declared.

